# Research dissemination workshops: observations and implications based on an experience in Burkina Faso

**DOI:** 10.1186/s12961-017-0205-9

**Published:** 2017-06-02

**Authors:** Esther Mc Sween-Cadieux, Christian Dagenais, Paul-André Somé, Valéry Ridde

**Affiliations:** 1 Department of Psychology, University of Montreal, P.O. Box 6128, Centre-ville Station, Montreal, QC H3C 3J7 Canada; 2 Action-Governance-Integration-Reinforcement/Health and Development Work Group (AGIR /SD), Ouagadougou, Burkina Faso; 3 School of Public Health, University of Montreal (ESPUM), 7101, Avenue du Parc, 3rd Floor, Montreal, QC H3N 1X9 Canada; 4 University of Montreal Public Health Institute (IRSPUM), Montreal, Canada

**Keywords:** Knowledge translation, Dissemination workshop, Evaluation, Research utilisation, Research use, Malaria, Global health, West Africa

## Abstract

**Background:**

In Burkina Faso, malaria remains the primary cause of healthcare use, morbidity and child mortality. Therefore, efforts are needed to support the knowledge transfer and application of the results of numerous studies to better formulate and implement programs in the fight against the malaria pandemic. To this end, a 2-day dissemination workshop was held to share the most recent results produced by a multidisciplinary research team. The objective of the present study was to evaluate the workshop and the policy briefs distributed there, the effects these produced on research results use and the processes that facilitated, or not, the application of the knowledge transmitted.

**Methods:**

A mixed-methods design was used. The data were drawn from a quantitative evaluation questionnaire completed after the workshop (*n* = 25/31) and qualitative interviews conducted with the researchers and various actors who attended the workshop (*n* = 11) and with participants in working groups (*n* = 40) that later analysed the policy briefs distributed at the workshop.

**Results:**

The participants recognised the quality of the research results presented, but felt that more needed to be done to adapt the researchers’ language and improve the functioning of the workshop. The potential effects of the workshop were rather limited. Effects were mainly at two levels: individual (e.g. acquisition of new knowledge, personal awareness raising) and local (e.g. change of practice in a local non-governmental organisation). Most participants perceived the utility of the research results, but several reported that their narrow decisional power limited their ability to apply this knowledge.

**Conclusions:**

This study showed the importance of workshops to inform key actors of research results and the need to undertake several different activities to increase the chances that the knowledge will be applied. Several recommendations are proposed to improve knowledge translation approaches in the West African context, including organising working and discussion groups, developing an action plan at the end of the workshop and offering support to participants after the workshop, among others.

## Background

Research results can be used to improve policy decisions about programs and services provided to the population [1, 2]. The continuing gap between available knowledge and its application is recognised as a reason for the persistence of poor population health indicators in low- and middle-income countries [3, 4].

In Burkina Faso, malaria remains the primary cause of healthcare use, morbidity and child mortality [5]. Numerous studies have been conducted on malaria in this country over many years. Nevertheless, national programs still have not benefited from their results because of the persistent reality that applying the knowledge produced by these studies is too difficult or simply not being performed [6, 7]. Thus, locally produced research results on malaria are perceived to have little impact on the decision-making process in the country [8].

Since 1991, a national anti-malaria program has been implementing interventions for both malaria prevention and treatment. In 2010, Burkina Faso received major funding of €63 million from the Global Fund to Fight AIDS, Tuberculosis and Malaria for this program [9]. A research program conducted as a partnership between Burkina Faso and Canada evaluated the implementation and effects of this program in the zones covered by the Kaya Health Observatory and its extension in Zorgho [10]. A second component looked at the local epidemiology of malaria and its effects on prevention and treatment strategies among children under 5 years of age [11]. In Burkina Faso, as elsewhere, health observatories produce very significant data for improving local health systems [12, 13]. However, these data are minimally used by decision-makers at the community or district level in low-income countries [14].

This research project attached particular importance to translation and application of knowledge. Besides producing and distributing policy briefs (PBs) and organising research dissemination workshops, the project recruited and trained a local knowledge broker to act as an intermediary between the researchers and potential users. This broker’s role was to foster optimal use of research results by adapting the knowledge to the target publics and creating opportunities for meetings to facilitate appropriation of new knowledge by actors. However, since the knowledge brokering strategy was at its beginning during the anti-malaria research project, the broker did not have an active role in this workshop. More information on the strategy development and implementation can be found in published articles [15, 16].

### Knowledge translation (KT)

KT is concerned not only with the dissemination of research results, but especially with their use and application. The concept of KT is defined by the Canadian Institutes of Health Research as “*a dynamic and iterative process that includes synthesis, dissemination, exchange and ethically-sound application of knowledge to improve the health, provide more effective health services and products and strengthen the health care system*” [17].

KT strategies that encourage interactions among the actors (e.g. seminars, training sessions, workshops, dissemination sessions) are preferable to KT strategies that are only informative (e.g. written reports, websites). These interactions are often seen to be a key determinant of research use [18, 19]. With interactive approaches, participants are more able to acquire new knowledge, share their experiences and develop new practices together [20].

Organising dissemination workshops is a KT strategy often used by researchers to disseminate their research results to potential users [4]. These workshops serve both educational and informative purposes, enabling researchers to transmit their knowledge in ways that are tailored towards a target public [17]. They also have an interactive component, as participants can express their own views and knowledge related to the research data [21]. In Burkina Faso, an evaluation has shown the utility of dissemination workshops for sharing research results [22].

### Organisation of a dissemination workshop

To present the preliminary results of the malaria research program to the main stakeholders, the researchers organised a 2-day workshop in November 2013. The objective was to inform the decision-makers from the two health districts concerned (districts being the smallest territorial unit of planning), leaders of the national program and the main actors involved in implementing anti-malaria interventions in the Kaya Observatory coverage zone. It was hoped that these data would be used in planning and implementing the anti-malaria program at the local and national levels to achieve better outcomes. A total of 31 people attended the 2-day workshop.

The workshop program, prepared and presented by the researchers, included 16 presentations of 15–30 minutes duration. The workshop’s main themes are listed in Table 1. Ten PBs summarising the presented results had been prepared by the researchers and were distributed at the workshop. These PBs were intended to provide key messages to be retained as well as recommendations.

**Table 1:** Main themes covered during the workshop on anti-malaria interventions

	Themes	Specific content
Day 1	Research program’s description	Presentation of the Kaya health observatory: research and evaluation
		Implementation and effects of anti-malaria interventions: research objectives
	Component 1: Implementation of malaria control interventions	The malaria control program in Burkina Faso has been well implemented but limited coverage and delays may have limited its effectiveness
		Community health workers’ perceptions of their involvement in malaria management are conducive to good performance
	Component 2: Effects of malaria prevention and treatment interventions	Rapid diagnostic tests for malaria are useful but are not always available or considered for diagnosis and prescription
		The availability of malaria rapid diagnostic tests has not led to a change in prescribing practices
		Children under five are rarely referred to a community health worker when they have fever
		Households needs have not been met by the 2010 distribution campaign of impregnated mosquito nets
Day 2	Component 3: Local epidemiology of malaria and intervention strategies	Prevalence and spatio-temporal concentration of malaria in Kaya district
		The practical implications of micro-territorial variations in malaria transmission
		The concentration of sick children in families and their involvement in the screening and treatment of siblings
	Component 4: Health equity	Effects of free healthcare access
		Perception of quality and costs of care in a context of free healthcare access
		National health insurance and risk-sharing
		Knowledge brokering as part of a research program: a mixed study
		Leadership and performance of health workers: study protocol

### Objective of the study

Evaluative studies are needed to determine whether the research knowledge was, or was likely to be, used by the key actors targeted [23, 24]. Studies measuring the effects of KT strategies are still relatively rare [23], particularly in Africa [25]. The objective of this study was therefore to evaluate the implementation of the dissemination workshop, the effects observed following it and the processes that facilitated, or did not facilitate, the use of the research results, as perceived by the workshop participants and the researchers.

## Methods

This evaluation used a mixed convergent qualitative–quantitative design [26]. Data from the quantitative and qualitative data collections were triangulated to obtain the most information possible on the workshop. Convergences observed between the quantitative and qualitative results reinforced the conclusions and enhanced our understanding of the workshop’s utility and impact [27, 28].

### Quantitative data collection

An evaluation questionnaire was completed by 25 of the 31 participants (81%) in attendance at the end of the workshop. It covered four components, namely (1) participants’ expectations; (2) their assessment of the workshop’s objectives, content and organisation; (3) their opinions regarding the utility of the knowledge acquired and their intention to use it to improve their practices; and (4) their suggestions for improving future workshops. The section on intention to use the knowledge was drawn from the questionnaire developed by Boyko et al. [29]. Statements were scored on a 7-point Likert scale from 1 (strongly disagree) to 7 (strongly agree). Data from the questionnaires were analysed using descriptive statistics.

### Qualitative data collection

#### Follow-up interviews

In the weeks and months following the workshop, 11 semi-structured follow-up interviews were conducted with seven participants and four researchers. Participants were selected using a contrasted sampling technique, which is intended to include at least one representative of each of the relevant groups of actors [30]. To ensure all views were included in the evaluation, interviews were conducted with respondents involved in decision-making and public administration (*n* = 2), program management (*n* = 1), non-governmental organisations (NGOs) (*n* = 2), health professions (*n* = 1), civil society organisations (CSO) (*n* = 1) and the research project (*n* = 4). Topics covered were their assessment of the activity and the PBs, the knowledge they had acquired, their intention to use that knowledge and how the workshop might be improved.

#### Group discussions on the PBs

Five of the 10 PBs (50%) were analysed in depth by another group of participants at a KT training session held several days after the workshop. This training was aimed at different actors in the West African health sector, including advisors and program heads in several ministries, physicians and researchers (*n* = 40). Even though they were not stakeholders in the dissemination workshop, their views as experts on the health system in Burkina Faso and in West Africa were useful for our analysis of the PBs. These participants, organised into subgroups of eight people, were asked to give feedback on the PBs in response to a set of questions (Was the language used appropriate? Was enough information provided? Was all the information relevant? Were the recommendations of value?) and the results were then discussed in a plenary session.

The interviews and PB analysis were audio-recorded with the consent of participants; the recordings were then fully transcribed and analysed using NVivo 11 software. The transcripts were thematically analysed, and a continuous thematisation process was used to conserve the richness of the body of data [31]. Themes were created and grouped over the course of the process to create categories. The main types of research-based knowledge use served as dimensions for analysing the workshop’s effects, namely conceptual or indirect use (changing one’s understanding or opinion on an issue), instrumental or direct use (changing one’s practices or decision-making), and persuasive use (influencing decisions, legitimising positions or actions, convincing others to adopt a position) [32].

## Results

### Dissemination workshop

The participants were very satisfied with the workshop content (5.6/7). While they recognised the utility of the knowledge presented (6.3/7), the overall score on intention to use it was relatively low (4.9/7) (Table 2).

**Table 2:** Overall average scores of participants (*n* = 25) for each component of the questionnaire

	Mean	Minimum	Maximum	Standard deviation
Objectives and workshop content	5.63	1	7	1.32
Organisation of the workshop	6.29	2	7	1.23
Utility of the knowledge	6.30	2	7	1.48
Intention to use the knowledge	4.88	1	7	1.54

The interview data provided more detail and substantiated these results. Table 3 compares the qualitative feedback from researchers and participants on each of the themes evaluated in connection with the dissemination workshop functioning. The themes were workshop content, accessibility, composition of the group of participants, perceived interactivity and recommendations proposed by the researchers based on research results. The main areas of convergence between the participants’ and researchers’ evaluations are described in the following paragraphs.

**Table 3:** Comparison of researchers’ and participants’ evaluations

Researchers	Participants
Content	
- Order of presentations logical, but some repetitions - Presentations conveying key message (e.g. lack of integration of anti-malaria interventions, community health workers (CHWs) not much used in urban settings, re-emergence of malaria in certain points of concentration identified each year, etc.)	- Results seen as credible, good quality of studies - Presentations by international researchers seen as sensitive to conditions in the field and adapted to the context - Scientific process too detailed for the non-initiated, but appreciated by those with research experience
Accessibility	
- Language used by researchers not always comprehensible to everyone - Anthropological content perceived as being accessible and interesting for participants - Geographic content on prevalence zones too complex and not presented in sufficiently simplified language	- Language comprehensible for actors in the health system - Language too specialised for public administration actors and local health workers - Presentations on prevalence zones too complex to understand
Group membership	
- Desire to have partners with decision-making power in attendance (regional and district-level decision-makers, anti-malaria program heads) - Adapting presentations challenging due to the diversity of actors in attendance (public administrators, nurses working in the field)	- Diversity of actors in attendance appreciated (researchers, non-governmental organisations, practitioners) - Absence of decision-makers from the central level perceived as a limitation - Concern that no CHWs attended the workshop
Interactivity	
- Discussions seen as helpful in delineating the problems - Hesitation by participants from the field to express themselves due to presence of decision-makers (public administration, anti-malaria program heads) - Hierarchical relationships potentially inhibiting any challenging of results presented	- Friendly environment that encouraged feedback from participants - Participants self-conscious and hesitant at first, but still able to present their views - Space created in which everyone’s (nurses, physicians, practitioners) concerns and opinions could be shared
Recommendations	
- Difficult to formulate recommendations geared toward specific actors - Perceived lack of receptivity and interest with regard to some proposed recommendations	- Recommendations and proposed solutions to problems both seen as being clear - Lack of concrete discussion on actions to be taken going forward

Source: Individual interviews with workshop participants and researchers (*n* = 11)

#### Accessibility of the content and of the researchers’ discourse

Participants recognised the quality of the studies presented and appreciated the process undertaken by the researchers to understand local conditions. However, several respondents indicated that they were more interested in the results and their practical implications on the ground than in the scientific process. Several participants said the language used by the researchers in their presentations was not sufficiently comprehensible to people working in public administration or to front-line workers. They considered that some researchers had used terms that were overly technical. Some respondents pointed out that methodological demonstrations could inhibit the understanding of participants who had no prior experience or training in research. Several researchers also acknowledged that some of the research content was difficult to convey in simple terms during the workshop, and that this might have caused confusion among some participants. This issue had, in fact, been raised in discussions among the researchers before the workshop, but it appeared not to have been taken into account by everyone.

#### Diversity of participants and group dynamics

Respondents appreciated the diversity of attendees at the workshop. However, several participants expressed surprise at the absence of community health workers, given their key role in implementing anti-malaria interventions. Several also expressed disappointment that no high-level decision-makers had attended, despite having been invited.

In general, neither the researchers nor the participants questioned the importance of disseminating research results not only to decision-makers and those in charge of anti-malaria programs, but also to NGOs in the field and to health workers. However, the format of the dissemination workshop as a ‘scientific conference’ at which the researchers made a series of presentations was seen as off-putting to certain target publics. One researcher suggested this format would need to be reconsidered if the objective was to bring all the different actors together at the same time.

Most participants said they had expressed their concerns during the discussions and that the climate was friendly and conducive to interactions with the researchers. For example, one participant noted that the presence of all the stakeholders made him more aware of the importance of coordinating the efforts of everyone involved in the fight against malaria:

“*I saw physicians who were there… there were NGOs, associations, and even mutualists* [mutual insurance managers] *attended, and all this wide range of experiences helped us to understand that this meeting was needed for us to share certain aspects…. There are elements we can agree on to better coordinate our activities.*” (NGO manager)

This diversity made it difficult, however, for researchers to adapt the content of their presentations:

“*The attendees were really diverse, so it was difficult to target the message…. the person who is a nurse in a health centre isn’t interested in the same message as the national anti-malaria program planner.*” (Researcher)

Even though several participants considered that the meeting had been useful for sharing views, the researchers noted that hierarchical relationships among the participants might have inhibited expression among some of them. The following excerpts clearly illustrate this difference in perceptions:

“*With the workshop, we understood that bringing these different actors together meant everyone has a role to play in achieving the objectives we are all pursuing.*” (NGO manager)

“*There were probably some representatives who were too high up compared to others, such that the ‘little guys’ didn’t dare say too much…. That’s something we see often in Burkina and elsewhere.*” (Researcher)

#### Clarity and application of researchers’ recommendations

Certain divergences were also noted in respondents’ views on the recommendations put forward for improving the anti-malaria program. The researchers continued to wonder whether their recommendations had been formulated clearly enough and whether they had actually reached their target audience, whereas the participants generally found the recommendations to be clear and comprehensible. The latter added, however, that they would have needed some time during the workshop to coordinate the actions that could potentially be implemented:

“*Everyone spoke and presented their views… but concretely, in relation to the results we got, what are we going to do?*” (NGO manager)

It is also possible that participants understood the relevance of the recommendations but did not have a clear vision of how they might be applied concretely on the ground, for example, with communities, in health centres and within the health system.

Additionally, while several participants recognised the importance of the recommendations proposed by the researchers, they described several barriers to their application. Obstacles to this application included the fact that programs were planned in advance and that resources were limited, and most respondents had no control over such programming:

“*There is evidence at the international level showing that treating children under five with preventive antimalarials during the winter period can reduce cases of malaria by more than 75%. Since 2012, we have had a well thought out action plan and have been looking for resources to implement it. So far, there are none.*” (Public administration manager)

The strong influence of international agencies that fund programs was also identified as a barrier by one researcher. Some researchers also perceived a lack of interest in their recommendations on the part of certain decision-makers.

### PBs with research summaries

In the individual interviews, those who had attended the workshop reported positive recollections of the PBs but were unable to cite details because of the time elapsed between the workshop and some of the interviews (up to 7 months). Several participants reported having read all the PBs (NGO and CSO representatives, health workers), while others only looked over some of the documents while at the workshop (decision-makers, public administrators).

In-depth analysis of the PBs in group discussions among participants at a KT training session a few days after the workshop identified areas for improvement (Table 4). In their view, the language was not accessible and several of the terms used were overly technical. Titles were often too long, complex and not clear enough. There were too many tables and figures presenting results, many of which were not considered helpful. Participants sometimes had difficulty understanding the aims of the PBs. Finally, most of the recommendations in the PBs did not propose concrete actions but instead were too general.

**Table 4:** In-depth evaluation of five policy briefs (PBs)

	Adapted language	Sufficient information	Relevant information	Recommendations
PB.1 Rapid diagnostic test (RDT) for malaria	Content accessible enough, but many technical terms	Ambiguous statements and many assertions without supporting data	Much of the information could have been removed	No clear, comprehensible and action-oriented recommendations
PB.2 Community health workers’ (CHWs) perceptions of their involvement in malaria management	Language not adequately adapted, overly technical and variable concepts	Insufficient information, title too long and unclear, many ambiguous statements	Many unnecessary tables	No clear recommendations, but a conclusion
PB.3 Management of children under five by CHWs	Target public not specified, making it difficult to assess whether language is adapted	Difficult to grasp the objective of the study	Difficulties in understanding what message the researchers were trying to convey	No clear recommendations, but a conclusion (presented as a question)
PB.4 Rapid diagnostic tests and prescribing practices	Language not adapted; long sentences and complex title	Insufficient information to understand the objective	Graphics difficult to understand; several unnecessary tables	Inconsistency between recommendations and study objectives
PB.5 Implementation of the anti-malaria program	Technical terms difficult to understand	Sufficient information	Some information not very relevant for the policy; table and figures improperly positioned in the text	No presentation of the key arguments at various points

#### Proposed improvements to the PBs

To improve the PBs, participants advised using simple language and short sentences, and providing more guidance to the reader on the actions to be undertaken and to whom they should be targeted. One participant also spoke of the importance of emphasising the advantage to decision-makers of using this knowledge (e.g. How would reducing the use of artemisinin combination therapies to treat malaria save money and time?).

For most of the researchers, this was their first experience writing PBs. Moreover, even though they had been offered the support of a KT specialist to adapt the content, few of the researchers availed themselves of this resource. Afterwards, several of them admitted they had difficulty using accessible and comprehensible language. Those researchers who had to write about more technically complex subjects (such as using spatial statistics to detect points of malaria concentration) found it even more difficult to convey their message clearly.

These researchers recognised the importance of focusing content on the actions to be undertaken, even though they were more accustomed to describing and explaining the methodologies of studies. Moreover, some felt it was important to emphasise the concrete utility of the results. If they had to do it again, they said, the PBs should be constructed around a key message and presented in terms of actions to be implemented by specific actors. According to this researcher, PBs should be conceived differently:

“*What do you want people to remember? What things do you want to see changed? It really means reorganizing the policy brief around* [a key message]*. We wrote them according to what was basically a scientific format: introduction, methodology, results… That’s not what a policy brief should be.*” (Researcher)

### Effects of the workshop on knowledge use

As mentioned earlier (Table 2), the participants saw the utility of the knowledge presented at the workshop (6.3/7), but their intention to use it was less certain (4.9/7). Table 5 presents the details of the scores for different items in the questionnaire component on intention to use the knowledge. On average, participants disagreed with the idea that using the knowledge was outside their control (2.7/7), but they were unable to assert that the decision to use the results depended entirely on them (4.7). The high standard deviation for this item may be due to the fact that the participants occupied positions with different levels of decision-making power.

**Table 5:** Levels of agreement among respondents (*n* = 25) on intention to use

	Min	Max	Mean	Standard deviation
The decision to use research data of the type presented at the workshop is outside my control	1	6	2.68	1.492
The decision to use research data of the type presented at the workshop depends entirely on me	1	7	4.00	2.330
I feel social pressure to use research data of the type presented at the workshop	1	7	4.17	1.775
People who are important to me in my professional life want me to use research data of the type presented at the workshop	1	7	5.09	1.276
I am expected to use research data of the type presented at the workshop	1	7	5.17	1.557
I am convinced I could use research data of the type presented at the workshop	2	7	5.77	1.110
I already see an opportunity where I could use all or some of the research data discussed in the workshop to help me in my work	5	7	5.96	0.706
I intend to use all or some of the research data discussed in the workshop to help me in my work	3	7	6.04	1.042

The qualitative data revealed a gradation in the effects of the workshop (Table 6). Several participants had relayed and re-disseminated the research results or the PBs in their organisation (persuasive use) after the workshop. For some, the results had confirmed what they already knew and believed (persuasive use). The workshop also provided new knowledge (conceptual use), as described by this health worker who learned important information on rapid diagnostic testing:

**Table 6:** Main effects reported by participants after the workshop

Type of knowledge use	Examples of knowledge use reported by participants
Knowledge dissemination (persuasive use)	- Preparing a report after the workshop for one’s superiors - Reporting to the members of a civil society organisation - Discussing the results at a meeting in a health centre - Loaning the policy briefs to colleagues to make them aware of the results
Confirmation (persuasive use)	- Confirming observations made by participants in the field (improper use of rapid diagnostic tests (RDTs), doing more than just distributing mosquito nets, etc.) - Confirming a belief that there was not a rise in prescriptions after the introduction of free healthcare
Learning (conceptual use)	- Learning about the researchers’ data collection process - Learning about the existence of research projects on malaria in Kaya and about the results - Theoretical learning (e.g. a positive RDT is required for each malaria case treated, free healthcare is not effective everywhere, household behaviours affect malaria propagation) - Results inspiring new ideas: following the example of the researchers’ data collection methods to send more workers into the field
Change (instrumental use)	- Reorienting certain interventions to better achieve the objectives - Individual awareness-raising: ensuring each patient has his own mosquito net - Changing practices in some health centres: waiting 20 days before repeating a treatment if the RDT is positive - Conducting home visits to verify the use of mosquito nets

Source: individual interviews with workshop participants (*n* = 7)

“*There was one professor who said that even when we do an RDT* [rapid diagnostic test] *and it’s positive, and then we do the treatment, if we redo the RDT 20 days later, it will still be positive. I didn’t have this information; I learned it at the workshop.*” (Health worker)

Several respondents also reported changes in their practices because of the workshop (instrumental use). One participant explained the change undertaken by his organisation:

“*It helped us a lot, because now when we hand out mosquito nets, we insist on these aspects to make sure the nets we give out are used full-time…. Now we do follow-ups; we’ve been doing them since the workshop.*” (CSO manager)

Finally, some reported that they intended to share or to use the research results in the near future, such as certain public administration managers who planned to present the results at upcoming district council meetings.

Participants who had relayed the PBs or workshop conclusions in their organisations were the ones who identified changes in their practices. These were local actors working in an NGO, a health centre or a CSO. On the other hand, decision-makers, whose positions were less operational, only mentioned their intention to report the results at upcoming district council meetings. Some sought the assistance of the team’s knowledge broker to come and present at these meetings.

The researchers speculated that dissemination workshops on their own cannot have any real impact, especially in terms of influencing public policies. In fact, the decision-makers did not follow-up with the researchers in the weeks following the workshop. The researchers wondered whether they had succeeded in conveying their message or whether this meant the actors were not interested in their ideas. According to these researchers, dissemination workshops help raise actors’ awareness of certain issues, but represent just a first step in KT.

### Suggestions for improving research use

Respondents made several suggestions for improving the organisation and follow-up of future workshops.

The knowledge transferred at workshops should be presented in ways that highlight their practical utility, along with cost-benefit data regarding their application. Some pointed out that actors first need to be convinced of the value of the knowledge before they will apply it. In terms of factors related to the KT strategy, a dissemination workshop should be held at an appropriate time such as when national programs are being developed. Strategic analysis is also needed to target potential knowledge transmitters. One researcher suggested inviting the ministries’ technical advisors, rather than high-level authorities.

A knowledge broker could also play the role of transfer agent to support participants after the workshop. According to the participants, a broker would be useful to provide information as needed by the actors:

“*It’s a job that could be very helpful to us, because we, as health workers out in the field, really don’t have time to look at research or its results, while there might be some very interesting results that could be useful in our everyday work.*” (Regional health department representative)

Respondents noted several obstacles related to the actors’ organisational contexts. However, these obstacles were outside the scope of a dissemination workshop. Frequently cited were the lack of financial, material and human resources for health interventions and the difficulty of obtaining funding to implement evidence-based programs. Additionally, as this participant explained, the health system’s organisational structure limits local workers’ decision-making power:

“*So, at the local level, we health workers can’t take decisions on anything at all related to malaria prevention until the central level has decided.*” (NGO manager and health worker)

Along these lines, some participants suggested that involving practitioners in the decision-making process would facilitate the appropriation of new knowledge or practices:

“*It’s important that everyone is involved in deciding about the intervention… rather than having the physician-in-chief who says, no, from now on, given the surveys that came out, this is what I’m deciding. Well, people won’t understand…. Together, we find a solution and take a decision.*” (Program manager)

Finally, several post-workshop actions were proposed to facilitate the application of knowledge transmitted, including presenting the results in national forums, using district chief medical officers to foster KT, presenting the results at statutory quarterly meetings with health workers and organising awareness-raising sessions in the field with health workers and the general public (e.g. on the importance of rapid diagnostic tests). In addition, to reach the general public, there were many suggestions involving the use of mass media (e.g. radio, television, advertisements, newspapers) and theatre forums. These various KT strategies could be used to disseminate knowledge in appropriately adapted formats.

## Discussion

The dissemination workshop was appreciated for the quality of the research results presented, but efforts had to be made to adapt the language and improve the functioning of the workshop. Moreover, the 10 PBs that were produced were not readily understandable to all participants and were not much used after the workshop. Even though the main types of knowledge use were reported by some respondents, the dissemination workshop had a limited impact overall. The principal effects reported were at the individual (acquiring new knowledge, personal awareness-raising) and local levels (practice changes in a local organisation, such as an NGO or CSO).

In light of the results of this evaluation, questions persist about certain policymakers’ lack of interest in research or in the work of researchers in general [18, 33, 34] given their absence at workshops or lack of follow-up afterwards. This point underscores the potential of research co-production (via participatory or collaborative research projects) to facilitate the uptake of research findings [19, 35, 36]. Involvement of stakeholders in position to use the research findings in the research process could permit a better match between research and stakeholders’ needs as well as a better knowledge appropriation due to their participation in the production. Hence, the policymakers’ lack of interest in research observed in this study could have been countered by greater engagement during the overall research process. The whole research program was developed in collaboration with stakeholders on the field since its inception in 2011–2012 [15]. Even if stakeholders were involved in needs assessment and in research question development, some studies more technical within the research program were not conducted in close collaboration with stakeholders. Therefore, maybe more regular contacts and greater implication during the research process would have helped this dissemination effort.

The sparsity of effects observed after this workshop on the anti-malaria program again raises questions regarding the potential impacts of end-of-grant KT activities organised by researchers. These KT strategies do not appear to be the most effective for fostering use of research results by actors from outside the academic milieu [37]. Hence, we also recognised the importance of including potential research users when developing KT strategies (reviewing key messages, suggesting credible presenters, understanding the communications network, etc.). Here, it may have been helpful if policymakers had been included, rather than just local decision-makers and other actors in the field. Indeed, district or local decision-makers in this context often have limited control over decisions because of financial constraints [14].

Nevertheless, the participants recognised the utility of the results since they were able to name some concrete actions that could be taken. However, several of them noted that their narrow decision-making power limited their potential to use the knowledge. Even though intention to use is a good predictor of use [29], other factors, often organisational in nature, influence the change process. More research is needed on the methods to influence practices and policies as well on what key actors should be involved in KT processes. Given the pyramidal structure of Burkina Faso’s health system, it is difficult for researchers to access the right people and to discuss matters with these intermediaries. Studies have shown the importance of understanding the organisation and functioning of social networks when planning KT strategies in Burkina Faso [38, 39].

A recent scoping review identified several factors to be considered when conducting KT processes in low-income countries [25]. Of the studies reviewed, the majority had evaluated KT strategies that targeted practitioners and the general public. The dissemination workshop evaluated in the present study had an equally political aim. The study’s results thus augment the list of factors compiled by Siron et al. [25]. Five conditions could improve research use in this context: (1) credible and useful knowledge providing clear orientations for action; (2) strategies targeting actors who are motivated and influential in their milieu; (3) strategies organised at the appropriate time to guide decisions; (4) transfer agents who hold strategic positions; and (5) organisations that involve health personnel in decision-making processes.

According to the results of the present study, certain key elements were not taken into account in organising this workshop on the anti-malaria program such as the notion of interactivity and sharing of experiences among participants. In this respect, the framework provided by deliberative dialogues, a KT strategy whose use is growing, would be useful for optimising knowledge application. A deliberative dialogue is a participative decision-making process used primarily to influence policies [40–42]; it recognises the plurality of actors’ knowledge and is used to create an egalitarian dialogue among all those affected, directly or indirectly, by an issue. These two features are essential for improving on traditional dissemination activities [43, 44]. Deliberative dialogues are more action-oriented. As such, a major portion of the exercise would involve participants deliberating on several aspects such as the issue under consideration, proposed solutions and potential obstacles to implementing those solutions [45].

A deliberative dialogue concludes with a series of commitments that the stakeholders intend to fulfil [46]. This method could improve dissemination workshops by emphasising the importance of the actions that the participants have committed to performing after the workshop. In preparation for a deliberative dialogue, one or more PBs are distributed at least 2 weeks beforehand to provide information so that the discussions will be based on research results, which was not done for the dissemination workshop analysed in the present study. These PBs summarise the main research results regarding the issue to be discussed as well as ideas for solutions [40, 47]. The PBs should be accessible to the audience targeted by the deliberative workshop, and scientific jargon should be avoided.

Studies have increasingly demonstrated the limited effectiveness of a KT process that is unidirectional, from researchers to potential users [41, 42]. Interactive strategies, which create reciprocal relationships between researchers and potential users, are often cited as being more effective in promoting knowledge use. However, such interactivity remains a difficult concept to operationalise, and because of this, studies have thus far been unable to confirm how it actually fosters research use [48]. Deliberative dialogues nevertheless increase the level of interactivity by involving participants more directly. Experiences of deliberative dialogue are on the increase on the African continent and offer an interesting alternative to dissemination workshops. Involving all actors in deliberations appears to be a good approach to foster research use and produce change [41, 49, 50]. However, more research is needed on this subject. The present study is important and offers lessons that could help improve future KT strategies. The research team that organised the dissemination workshop in Kaya took careful note of these lessons; 2 years later, the team prepared a workshop in the form of a deliberative dialogue, and conformed entirely to the procedure [51], to discuss the results of several studies on road accidents.

Finally, a dissemination workshop should not be the end-point of a KT process. Support and additional strategies should be offered to participants following the workshop. Given participants’ enthusiasm for knowledge brokering, significant efforts should be made to involve a broker in the process. Even though this role is new in Burkina Faso, actors in the health field recognise its value [15]. For example, the broker can support knowledge application after the workshop, organise other follow-up workshops or meetings and provide the actors with more research results [16]. Unfortunately, while this intermediary role is primordial in low- and middle-income countries, it is often non-existent [52].

### Limitations of the evaluation

One of the main limitations is the small number of interviews conducted (*n* = 11). To strengthen the conclusions, at least two representatives of each group of actors would have been required, which was not possible in this case. Additionally, the elapsed time between the workshop and the follow-up was long for three participants (around 7 months), and some participants had difficulty recalling certain elements. It was therefore difficult to associate changes directly with the dissemination workshop. Given the lack of any follow-up, aside from the interviews, it was not possible to evaluate other possible effects of the workshop. Further, the questionnaire would need to be validated to ensure its reliability and sensitivity as a tool. As the majority of scores on the questionnaires were high, it may be that, in an aid-dependent country, a social desirability bias influenced participants to evaluate the workshop positively [53]. Evaluating the effects of KT strategies, including knowledge use, remains a challenge in this field [54]. Moreover, some effects, particularly at the public policy level, are only produced after several years [24]. Another limit to this evaluation is that data were restricted to the participants’ perceptions of effects. Thus, it is possible that some other impacts may have resulted from the anti-malaria research project, but we are not aware of any other effects than those presented. The gathering of evidence on any actual actions taken after dissemination (e.g. policies drafted) is a challenge in KT evaluation.

## Conclusion

This case study evaluated the utility of a dissemination workshop in fostering the use of research-based knowledge. Based on the results, certain recommendations can be made to improve the organisation of such workshops in Burkina Faso:

- Formulate a clear and well-defined objective: pose a clear question to which the workshop will attempt to respond and validate the objective of the workshop with the participants (e.g. decision-makers).
- Offer researchers the support of a KT specialist: adapt the presentation slides and PBs to eliminate scientific jargon.
- Pay special attention to participant recruitment: maximise the chances that the research results will be applied by analysing the actors’ social networks.
- Plan time for discussion among the participants: reduce the number of scientific presentations to organise more group work or guided discussions.
- Develop a post-workshop action plan: deliberate with participants on concrete actions to be taken after the workshop.
- Offer participants the support of a knowledge broker after the workshop: the broker can help with district meetings, organising workshops or training sessions, advocacy preparation, etc.

The present study reinforces that researchers need to continue striving to make their knowledge more accessible to foster its application. They must become more competent in addressing a non-academic public [55, 56]. The review conducted with the researchers after the workshop helped develop their reflexivity regarding their strengths and weaknesses in KT. Moreover, the advancement of our understanding of the mechanisms that foster KT in low- and middle-income countries will require process evaluation and follow-up with participants on their application of the knowledge acquired.

## Abbreviations

CSO: civil society organisation
KT: knowledge translation
NGO: non-governmental organisation
PB: policy brief
